# Progress in Surgery of the Stomach

**Published:** 1903-10-10

**Authors:** 


### Progress in Surgery of the Stomach.

(*Concluded from page* 17.)

**Gastro-enterostomy.**—Lloyd [12] reports seven cases treated by McGraw's method. In it the anastomotic aperture is not made at the time of the operation, the walls of the stomach and intestine being strangled by an elastic ligature, which takes two or three days to cut through the enclosed tissues, and so form a direct communication between the two viscera. The ligature used is a rubber cord, smooth, round, and with a diameter of two millimetres, one end of it being shaved thin so that it may be drawn through the eye of a so-called worsted needle for convenience in passing. This also has the advantage that it makes a smaller hole in the intestinal wall, and its subsequent retraction causes it to completely fill the orifice made in the wall of the viscus. The stomach and jejunum are first united for a distance of six or seven centimetres with a row of Lembert sutures. The ligature is then passed through the opposed walls of the two viscera so as to include in its grasp one inch or more of each. It is then tied tightly and the knot prevented from slipping by tying a silk thread firmly round it. A second row of Lembert sutures is then placed, uniting the visceral walls in front of the ligature and completely burying the knot. In two or three days the ligature has cut through and the edges of the orifice so formed are found closely united without any raw surface whatever. The advantages of this method are :—1. It is the quickest of all methods of intestinal anastomosis. 2. There is no incision in the bowel, no loss of blood, no escape of fæces, and no exposure to infection from the bowel contents. 3. The surgeon can govern exactly the length of the opening. Caird [13] reports 21 cases of gastro-enterostomy, four of which were fatal. Mayo Robson [14] uses a decalcified bone bobbin for the operation, and he considers that the different indications for gastro-enterostomy cannot be stated in one category, for while in the first series the indications, with certain provisos, are absolute so soon as the diagnosis is established, in the second series the indications for the operation apply only after failure of general treatment. In the first series are : — 1. Simple stenosis of the pylorus. 2. Malignant stenosis of the pylorus. 3. Congenital stenosis. 4. Congenital atresia. 5. Chronic gastric ulcer with tumour of doubtful character too extensive or adherent for effectual removal, not necessarily associated with stenosis. 6. Duodenal cancer or tumour causing obstructive symptoms. 7. Hour-glass contraction of the stomach not favourable for gastroplasty. 8. Perigastritis with adhesions around the pylorus producing obstructive symptoms or pain and too extensive for gastrolysis. 9. Dilatation of the stomach dependent on pressure outside the pylorus from tumour of the pancreas, liver, or gall-bladder incapable of removal. In the second series after failure of medical treatment are :—10. Acute or chronic gastric ulcer. 11. Duodenal ulcer. 12. Hæmorrhage from gastric or duodenal ulcer. 13. Persistent spasm of pylorus or Reichmann's disease with dilatation of the stomach. 14. Hyperchlorhydria. 15. Persistent gastralgia. 16. Tetany of gastric origin. 17. Acute gastric dilatation not yielding to lavage and general treatment. 18. Pancreatitis secondary to gastric ulcer. 19. Certain cases of jaundice dependent on gastric or duodenal ulcer leading to thickening around the common bile duct. 20. Simple atonic dilatation of the stomach after failure of lavage and general treatment.

**Stenosis of the Pylorus.**—Quenu and Petit [15] state that in 35 cases operated upon by different methods the mortality was 22·8 per cent. Three cases were treated by dilatation of the pylorus, with one death and two failures. In four cases resection of the pylorus was performed and each time the operation was a success. Twenty-three cases were treated by pyloroplasty, with five deaths, or a mortality of 21·7 per cent. In seven cases gastro-enterostomy was performed, with two deaths, or a mortality of 28·8 per cent. In view of all the facts the authors draw the conclusion that gastro-enterostomy is the best operation for the relief of stenosis of the pylorus. The posterior operation, with sutures, is the operation of choice. In cases where the stomach itself has been much altered by the swallowing of a caustic fluid and is small and shrivelled up, gastro-enterostomy is contra-indicated. In one such case Hartman performed duodenostomy. Finney [16] urges early operation in benign cases as well as malignant ones. Vicious circle was found in 38 out of 550 cases of gastro-enterostomy, and although many efforts have been made to obviate this the problem has not yet been solved. Rivière [17] reports a case of congenital hypertrophic stenosis of the pylorus, Anning [18] a case of malignant disease of the pylorus in a man aged nineteen years, and Symonds [19] and Thorburn [20] report other cases of obstruction.

Pyloroplasty by a new method is advocated by Finney.[21] The duodenum is laid by the side of the pyloric end of the stomach and a communication made between the two after the manner of lateral anastomosis suggested by Halsted. He urges the following points in favour of pyloroplasty :—1. Regurgitation of bile into stomach is prevented. 2. Secretion of hydrochloric acid, when it has been excessive becomes normal. 3. If the secretion of hydrochloric acid has been diminished or absent before the operation, it remains *in statu quo* after operation. 4. If there has been primary gastric atony, peristalsis is but little improved. 5. This function improves rapidly or reaches perfection if the muscular contractility has been normal or increased, and when the obstruction was due to fibrous stenosis or pyloric spasm. 6. In all such cases evacuation of the stomach is accomplished in its physiological period. Only in rare cases, and these only in the first months after operation, may it be delayed. 7. The capacity of the stomach always decreases, but rarely becomes as small as normal. 8. The pylorus recovers tone. The advantages in favour of his particular method are :— 1. The ease and facility with which it can be performed. 2. Its low mortality, 6·8 per cent. 3. Its success in at once relieving the disagreeable symptoms. 4. Its applicability, even in the presence of dense adhesions about the pylorus. 5. Except in cases of extreme dilatation it furnishes effective drainage, placing the outlet at or on a level with the most dependent portion of the stomach. 6. It absolutely prevents spur formation and the vicious circle. 7. It places the new pylorus in approximately its proper place.

**Hairballs** and other concretions in the stomach are discussed by Fenwick.[22] Out of 24 cases of hairballs no fewer than 23 were females, the youngest of whom was 18 and the oldest 34 at the time of death. There was never any indication of mental disease, and in several instances it was expressly stated that the patient was neither hysterical nor particularly emotional. A tumour is present in most cases of concretion, and this may be confused with an enlarged spleen, a floating kidney, or with a fæcal accumulation in the colon. If the tumour is small in size it may be possible to secure its removal by an emetic, but this method is always fraught with a certain amount of danger on account of the frequent co-existence of secondary ulceration of the stomach or duodenum. In the case of large tumours recourse must be had to gastrotomy. Other cases are reported by Morten,[23] Mallins,[24] and in the *Lancet*.[25]

[12] Med. Chron., March, 1903, p. 429, and Med. Rec., New York, Feb. 28, 1903, p. 351. [13] Amer. Jour. Med. Sci., Dec., 1902, p. 1091. [14] Lancet, Feb. 28, 1903, p. 570. [15] Amer. Jour. Med. Sci., Dec., 1902, p. 1093. [16] Med. Rec., New York, March 7, 1903, p. 396. [17] Lancet, Dec. 27, 1902, p. 1750. [18] Ibid., Nov. 22, 1902, p. 1386. [19] Brit. Med. Jour., Feb. 28, 1903, p. 482. [20] Med. Chron., Jan., 1903, p. 255. [21] Bulletin of the Johns Hopkins Hospital, July, 1902, p. 155. and Med. Rec., New York, March 7, 1903, p. 397. [22] Brit. Med. Jour., Nov. 29, 1902, p. 1696. [23] Lancet, May 16, 1903, p. 1373. [24] Ibid., June 6, 1903, p. 1592. [25] Ibid., p. 1606.

